# Long-term survival after anti-PD-1 discontinuation in advanced cutaneous squamous cell carcinoma (cSCC): a proof of concept of benefit of concomitant cemiplimab and radiotherapy

**DOI:** 10.1007/s00262-024-03700-x

**Published:** 2024-05-07

**Authors:** Barbara Bailly-Caillé, Romain Levard, Diane Kottler, Anne Dompmartin, Jean-Matthieu L’Orphelin

**Affiliations:** 1 Department of Dermatology, Caen-Normandie University Hospital, 14000 Caen, France; 2 INSERM ANTICIPE UMR1086, Interdisciplinary Research Unit for Cancer Prevention and Treatment, Normandie University, Research Building F-14000 François 3 Avenue Général Harris, BP 45026, 14076 Caen Cedex, France

**Keywords:** Cutaneous squamous cell carcinoma, Immunotherapy, Cemiplimab discontinuation

## Abstract

**Background:**

In a princeps study we conducted in patients with advanced cutaneous squamous cell carcinoma treated with concomitant anti-Programmed cell death protein 1 (PD-1) and radiotherapy, we demonstrated a clinico radiological response to cemiplimab that appeared to persist over time, 1 year after treatment discontinuation.

**Method:**

We conducted a single-center descriptive study at Caen Hospital from September 1, 2021 to September 2023, in 14 patients with advanced carcinoma treated with cemiplimab until September 1, 2021. The aim of this update is to examine clinical and radiological follow-up 2 years after discontinuation of cemiplimab.

**Results:**

Of the 12 patients with a partial or complete response, we report 8 (66.7%) persistent responses 2 years after stopping cemiplimab, with only 2 patients progressing to distant disease, one lost to follow-up, and one death a priori unrelated to the disease.

**Conclusion:**

Our study confirms a long-term and persistent effect despite discontinuation of cemiplimab at least up to 2 years later.

## Introduction

Advanced cutaneous squamous cell carcinomas (locally advanced and metastatic cSCC) are infrequent and have a worse prognosis than common primary cSCC. For those who are not candidates for curative surgery or radiotherapy, treatment with anti-PD-1 immunotherapy is first-line therapy gold standard (1). Numerous real-life studies have reinforced the place of cemiplimab, a monoclonal antibody targeting the PD-1, in the management of advanced cSCC (2, 3, 4, 5). In our princeps study, we showed a clinico radiological response to cemiplimab that seemed to persist over time (5). Following our original clinical study on the concomitant benefit of cemiplimab (5) and radiotherapy, we propose an update after two years of follow-up.

## Materials and methods

We performed a single-center descriptive study in Caen hospital from September 1, 2021 to September 2023, with evaluation of the clinico radiological response in 14 patients with advanced carcinoma treated with cemiplimab until September 1, 2021, when the cemiplimab was discontinued due to loss of reimbursement following the decision of the French transparency commission.

Patients were evaluated every 3 or 6 months, clinically and radiologically with PET tomodensitometry, cerebro facial MRI or thoraco abdomino pelvic scanner depending on tumor location; and according to the iRECIST criteria.

Data are expressed as the mean ± standard deviation and percentage.

The study complied with the ethical standards set out in the Declaration of Helsinki and was approved by the Ethics Committee of Caen University Hospital.

## Results

### Patients

We included 14 patients with advanced cutaneous squamous cell carcinoma and who were still undergoing treatment with cemiplimab in August 2021, date of discontinuation due to loss of reimbursement in our center.

Baseline patient characteristics are shown in Table 1. The majority of patients were male, aged over 75, in good general condition and with a history of cutaneous squamous cell carcinoma (57.1%). Patients mainly had metastatic disease (85.7%) with locoregional rather than distant metastasis (75.0 vs 25.0%).

**Table 1:** Patient characteristics

	(*n* = 14)
*Age (years)*	75.9 ± 12.1
< 65 yo	1 (7.2)
65–75 yo	3 (21.4)
> 75 yo	10 (71.4)
*Gender*	
Male	12 (85.7)
Female	2 (14.3)
*ECOG status*	
0	2 (14.3)
1	8 (57.1)
2	4 (28.6)
*Previous cSCC*	
No	6 (42.9)
Yes	8 (57.1)
*Immunodepression*	
No	10 (71.4)
Yes	4 (28.6)
*Lymphopenia*	
No	8 (57.1)
Yes	6 (42.9)
*Staging*	
LacSCC	2 (14.3)
mcSCC	12 (85.7)
Locoregional metastasis	9 (75.0)
Distant metastasis	3 (25.0)
*Site*	
Face	13 (92.9)
Scalp	1 (7.1)
*Previous lines of therapy*	
No	14 (100.0)
Yes	0
*Histological features*	
Degree of differentiation	
Well	7 (50.0)
Moderate	5 (35.7)
Poor	2 (14.3)
PNI	
No	7 (77.7)
Yes	2 (22.2)
Bone erosion	
No	10 (83.3)
Yes	2 (16.7)
Invasion beyond subcutaneous fat	
No	4 (36,4)
Yes	7 (63.6)
*Cemiplimab dosage*	
350 mg every 3 weeks	13 (92.9)
3 mg/kg every 2 weeks	1 (7.1)
*Intent of radiotherapy*	
Curative	5 (100)
Palliative	0
*Site of radiotherapy*	
Primary tumour	1 (20.0)
Metastasis	4 (80.0)
*Dose per fractions (Gy)*	2.6 ± 0.5
*Fractions*	25.7 ± 7.0
*Prescribed dose*	63.3 ± 8.6
*BED*	79.5 ± 10.8

Results are expressed as mean ± standard deviation or number (%). *ECOG* Eastern Cooperative Oncology Group, *cSCC* cutaneous squamous cell carcinoma, *La* locally advanced, *m* metastatic, *PNI* perineural invasion, *Gy* grey, *BED* biologically effective dose

### Clinical and radiological follow-up of patients

At the end of treatment, in August 2021, 14 patients were being followed at our center: 9 patients have a complete response, 3 a partial response, one patient had a stable disease and one patient had a progressive disease. Of these 14 patients, 5 received concomitant Cemiplimab and radiotherapy. None received a hypofractionated radiotherapy regimen. One initial SD-patient was lost to follow-up, and we report 1 rapid death of another patient initially PD. Of the 3 CR among these 5 patients treated with concomitant treatment regimen, 1 deceased at 6 months, and 2 were still in CR after cemiplimab discontinuation.

At 6 months, two patients had died (one with progressive disease and one with a complete response, a priori unrelated to the primary disease). One patient was lost to follow-up and showed a stable response when cemiplimab was stopped.

At 12 months of discontinuation to cemiplimab, the 8 patients in complete response had a persistent response, and only one patient in partial remission had a progression.

**Fig. 1:**
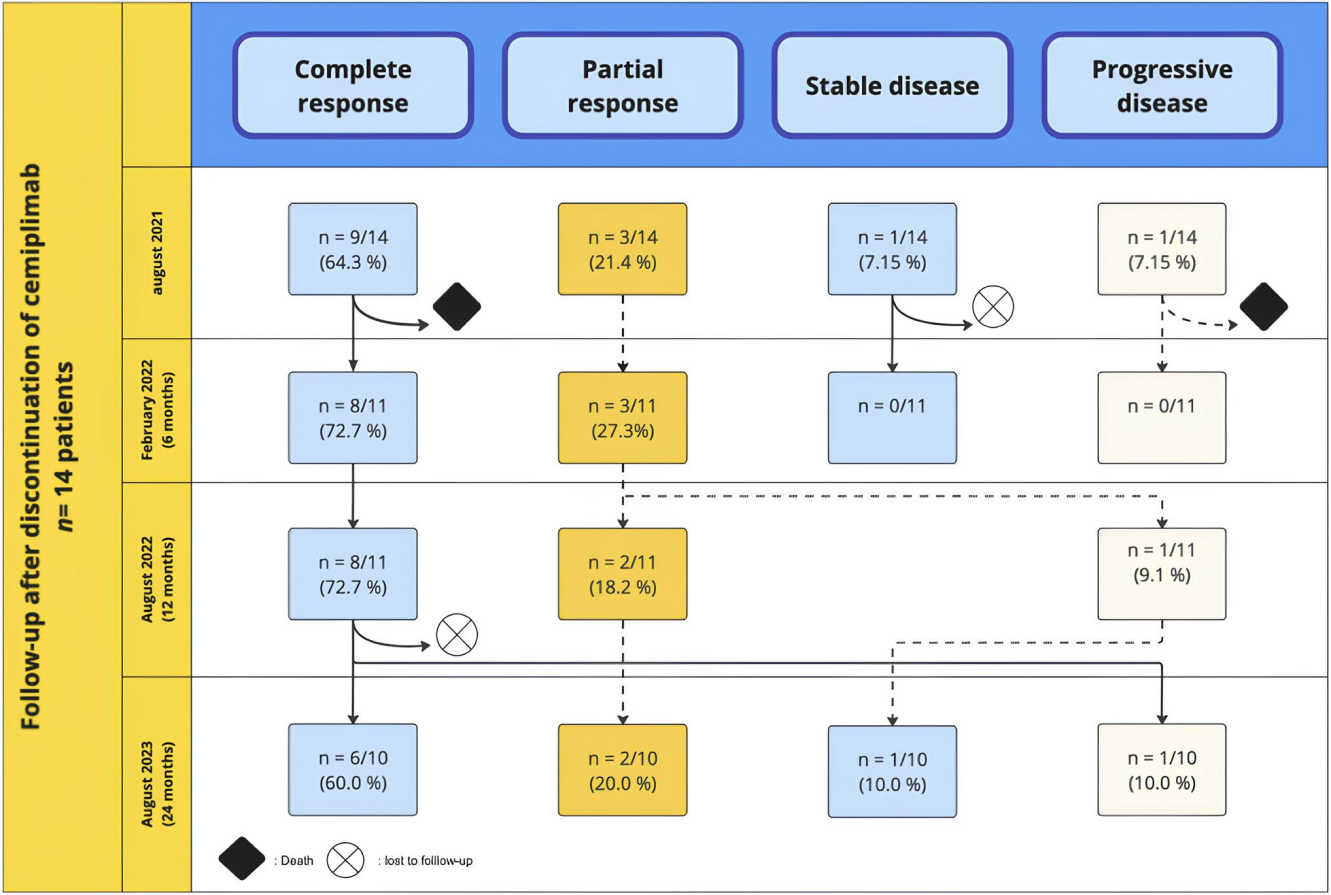
Follow-up after discontinuation of cemiplimab

Finally, at 24 months after stopping cemiplimab 6 patients had a persistent complete response: One patient with complete response was lost to follow-up and one patient have a progressive disease. The 2 patients with partial response had persistent response. The patient with progressive disease at 12 months has finally a stable disease at 24 months (Fig. 1).

## Discussion

There are few data on the discontinuation of immunotherapy, and in particular of cemiplimab, in the management of the cutaneous squamous cell carcinoma. Our study shows a long-term and persistent effect despite cessation of cemiplimab up to 2 years after discontinuation. Indeed, among the 12 patients presenting a partial or complete response, we report 8 (66.7%) persistent responses at 2 years after stopping cemiplimab, with only 2 patients having progressed at a distance, one lost to follow-up, and one death a priori unrelated to the disease. This persistent effect may be explained by a persistent immunogenic effect even after treatment has been discontinued. Interestingly, lymphopenia did not appear as a risk factor for progression in univariate analysis in our princeps article (HR 1.1 [0.4–3.6], *p* = 0.830) (5). Now two years after stopping treatment, 6/8 non-lymphopenic patients were still responding, compared with 2/6 lymphopenic patients. However, the clinical situation of the latter was more precarious at the time of treatment discontinuation, making comparisons difficult. Furthermore, it is not possible to discuss the benefit of the subgroup treated with cemiplimab and concomitant radiotherapy because the number of patients was too small (*n* = 5/14, with one patient lost to follow-up and one death). Others notable limitations of our study are its descriptive framework and the absence of a control group. This limits our ability to establish a definitive link between observed outcomes and treatment regimen. Similarly, we were unable to study the influence of cemiplimab dosage on the response observed, given the almost exclusive use of a 350 mg flat dose every 3 weeks.

Older patients are likely to respond to ICI with fewer adverse events than younger ones. In our study, over 70% of our patients were over 75 years of age, which seems to confirm the interest of anti-PD-1 for these elderly and polymorbid patients (6).

Furthermore, our results are consistent with the data from the final analysis of the EMPOWER-CSCC-1 study (7), which evaluated cemiplimab in the same reference population in terms of overall and relapse-free survival (RFS), as well as safety profile. This pivotal, non-comparative Phase II study was the basis for the European and American health authorities' validation of cemiplimab for the treatment of patients with locally advanced or metastatic cSCC ineligible for curative surgery or radiotherapy.

Cemiplimab's position as the treatment of choice in locally advanced or metastatic disease has been reinforced by data from the TOSCA study (8), the first comparative study of cSCC to assess its efficacy and safety compared with historical systemic therapies (HTS). This large French cohort was conducted on 28 sites. It demonstrated significantly longer outcomes for patients treated with cemiplimab compared to HTS.

ICI is now the gold standard, as since pembrolizumab, another anti-PD-1 agent, has been the subject of an encouraging phase 2 study, MK-3475 (9), in the metastatic setting, while also maintaining acceptable tolerability. Adjuvant immunotherapy is therefore a promising new approach in advanced cSCC with a high percentage of complete pathological response (10).

Although no consensus has been reached, in long-responder patients undergoing treatment with cemiplimab we could discuss spacing infusions rather than stopping them altogether, in order to maintain a long-term immunogenic effect, as has been proposed in Merkel carcinoma and melanoma (11). Further studies are needed to optimize the management of long responders treated with immunotherapy.

In any case, current European recommendations for follow-up after cessation of active treatment in locally advanced or metastatic disease (12) call for lymph node ultrasound every 3 to 6 months for 5 years, then every 6 to 12 months thereafter. Whole-body imaging (CT scan, MRI or PET scan) is also recommended every 3 to 6 months for 3 years, then adapted according to individual patient data.

Given the promising results of immunotherapy in the adjuvant setting, the question has arisen as to whether it would be useful in the neoadjuvant setting. The aim of this strategy is to reduce the tumor size prior to surgery, thereby reducing the surgical field and facilitating reconstruction. The phase 2 study by Gross et al. (10) shows interesting results with the use of neoadjuvant cemiplimab, with pathological complete response rates of 51% and major pathological response rates of 13%. Similarly, preliminary survival data appear encouraging, with an event-free survival at 12 months of 89%, an absence of recurrence in the group of 40 patients with pathological complete response, and an overall survival at 12 months of 92%, with a median not yet reached. The use of this neoadjuvant approach could save radiation doses for a future need, possibly combining radiotherapy and cemiplimab.

Radiotherapy is often used in the adjuvant setting in patients with poor prognosis factors, to reduce the risk of local relapse. Recently, Ruiz et al*.* (13) demonstrated a lower risk of locoregional recurrence than surgery alone. Adjuvant radiotherapy remains widely debated; currently available studies struggle to show a clear benefit of adjuvant radiotherapy, as evoked by the systematic review and meta-analysis conducted by Kim Y. et al*.* (14).

The efficacy of radiotherapy could be enhanced by concomitant use with an anti-PD-1 agent as shown in our princeps study since we reported a 2.5-month reduction in response time when combining radiotherapy (RT) and cemiplimab (C) (median response time was 5.5 months in the C group vs. 3 months in the C/RT group). Our update at 2 years after stopping cemiplimab shows a preserved long-term response. This suggests the persistence of the immunological response induced by cemiplimab against tumor cells, notably preservation of the cytotoxic function of CD8 + T lymphocytes. This effect is enhanced by concomitant radiotherapy (5) inducing immunogenic phenomena, by generating neoantigens and modulating the tumor microenvironment.

## Conclusion

Updated data from our study evaluating the benefit of radiotherapy performed concomitantly with cemiplimab in locally advanced or metastatic cSCC shows a long-term and persistent response after cemiplimab discontinuation with more than two years' hindsight. Our initial data had already shown that the combination of cemiplimab and radiotherapy achieves a faster objective clinico radiological response than with cemiplimab alone, as well as an improvement in local symptomatology, without increasing the occurrence of AEs. Updated data confirm the value of this combination therapy for advanced stage cSCC and long-term response, which needs to be confirmed in large-scale prospective studies.

## Data Availability

The datasets generated during and/or analysed during the current study are available in the research center of Caen.
